# A reinforcement learning-guided interpretable method for postoperative sepsis prediction with Hilbert-Schmidt Independence Criterion

**DOI:** 10.3389/fdata.2026.1811110

**Published:** 2026-04-07

**Authors:** Kunhua Zhong, Han Chen, Qilong Sun, Peng Wang, Zhenbei Liu, Yuwen Chen

**Affiliations:** 1 Chongqing Institute of Green and Intelligent Technology, Chinese Academy of Sciences, Chongqing, China; 2 Fuling District Linshi Community Health Service Center, Chongqing, China; 3 Centre for Medical Big Data and Artificial Intelligence, The First Affiliated Hospital (Southwest Hospital) of Army Medical University (Third Military Medical University), Chongqing, China; 4 Chongqing University Fuling Hospital, Gastroenterology, Chongqing, China

**Keywords:** feature engineering, Hilbert–Schmidt Independence Criterion, large-scale medical data, reinforcement learning, self-attention, sepsis, temporal convolutional networks

## Abstract

**Background:**

Sepsis is a major cause of postoperative morbidity and mortality, and early risk stratification from perioperative electronic health records (EHR) is a representative large-scale, high-dimensional data processing problem that requires models to be accurate, efficient, and clinically interpretable. However, many existing sepsis prediction methods operate as black boxes and rely on extensive temporal monitoring streams, which increases feature dimensionality and computation while limiting transparency.

**Methods:**

We propose a reinforcement learning-guided, interpretable feature engineering framework for postoperative sepsis prediction that targets scalable learning on heterogeneous perioperative data. Within an Actor-Critic formulation, feature selection is treated as an action: an Actor network produces a stochastic feature mask over preoperative static variables and intraoperative statistical summaries, while a Critic network performs downstream prediction using a self-attention-based classifier. To benchmark and stabilize learning, we introduce an auxiliary baseline model that incorporates intraoperative temporal signals extracted by a temporal convolutional network (TCN) and regularized using the Hilbert-Schmidt Independence Criterion (HSIC) to encourage non-redundant representations between statistical and temporal feature views. The Actor is optimized to achieve comparable predictive performance to the baseline while using a reduced feature set, improving computational efficiency and supporting instance-level interpretability.

**Results:**

Experiments on a real-world surgical cohort from Southwest Hospital (2014-2018) demonstrate that the proposed framework attains performance comparable to or better than competitive machine learning baselines while selecting fewer input features. On this dataset, our method achieved perfect scores of 1.00 for F1-score, Sensitivity, and Specificity.

**Conclusion:**

The proposed method accurately predicts the occurrence of postoperative sepsis and provides effective instance-level post hoc explanations. These findings offer a novel perspective for postoperative sepsis prediction.

## 1 Introduction

Sepsis is a life-threatening organ dysfunction resulting from a dysregulated host response to infection. Due to the complexity of its pathogenesis, the heterogeneity of clinical presentations, and the intricacy of treatment strategies, accurate prediction and effective decision support for sepsis remain enduring challenges in critical care and are recognized as a global health priority. According to 2022 estimates by the World Health Organization, the global burden of sepsis is substantial, with approximately 30 million cases annually and up to 6 million deaths, corresponding to a mortality rate as high as 20%. The situation is particularly severe in China, where the mortality rate reaches 36%. During the COVID-19 pandemic, it was estimated that nearly 60% of critically ill patients developed sepsis, further intensifying the public health crisis. The onset of sepsis is acute and severe, demanding immediate intervention; delays in treatment by just 1 h may increase mortality by approximately 6%. Despite its urgency and gravity, no optimal treatment or interpretable intelligent decision-support tool currently exists for sepsis beyond general clinical guidelines. Therefore, the development of early, accurate, and interpretable predictive tools for sepsis holds great promise in assisting healthcare professionals and effectively reducing sepsis-related risks.

At present, artificial intelligence (AI) is developing rapidly and has been widely applied in various medical fields, including clinical assistant diagnosis and treatment (Chen et al., 2021), prognostic prediction (Kaushik et al., 2020; Wong et al., 2020), surgical workflow identification (Zhao et al., 2023; Wang et al., 2025), medical image analysis and processing (Zhu et al., 2023; Xiao et al., 2023), pathologic study (Jiang et al., 2020), genetic analysis (Shao et al., 2023), clinical assisted decision-making (Singh et al., 2023; Eloranta and Boman, 2022), and so on. A large number of studies have harnessed AI for sepsis prediction and achieved high accuracy (Goh et al., 2021; Chen et al., 2023; Li and Wang, 2023; Wu et al., 2024; Tang et al., 2024). Previous researches mostly focused on the performance of models. Existing research results indicate that models based on deep learning are generally outperform experts or classic algorithms in medical field (Fan et al., 2021). However, due to the high-risk nature of medical applications, only a deep model that can produce the correct answers without explanation is insufficient. Current machine learning models employed for sepsis prediction are still black boxes, lack quality control and high-quality explanations. Consequently, the credibility of these models is diminished, posing a significant challenge in their application to guide actual clinical diagnosis and decision-making. Then, the main research question of our study is: Is there an interpretable method for accurately predicting postoperative sepsis?

Reinforcement learning (RL), a pivotal subfield of machine learning, is renowned for its formidable decision-making capability. In recent years, many important theoretical and practical achievements have been made in this field, which have been widely attempted in clinical decision support, as reviewed comprehensively by Chai et al. (2025). Beyond standard RL, its variants such as inverse reinforcement learning (IRL) have also shown promise in learning optimal control policies from expert demonstrations, as demonstrated in complex systems like Takagi-Sugeno fuzzy models (Song and Tong, 2025). However, in the field of sepsis, RL and its variants are mainly used for intelligent decision-making rather than prediction in the field of sepsis. RL itself has a certain degree of interpretability. If RL can be applied to the prediction problem of sepsis, it would be a promising idea.

Before building such a predictive model, a fundamental challenge lies in learning effective representations from heterogeneous medical data, which integrates static preoperative variables with dynamic intraoperative signals. Robust data representation learning, aiming to extract compact and informative features from these multimodal inputs, is crucial for improving both predictive accuracy and model interpretability. Although advanced methods like non-gradient hash factor learning have been proposed to handle high-dimensional and incomplete data (Wu et al., 2026), such techniques remain largely unexplored in the context of postoperative sepsis prediction.

Despite significant achievements of interpretability research works in the medical field, the vast majority of models for postoperative sepsis prediction are lack of interpretability. Therefore, to overcome the interpretability issues mentioned above, the main objective of this article is to construct an accurate prediction method with interpretability for postoperative sepsis using the Actor Critic framework of RL, and provide post hoc explanations.

In this paper, we applied reinforcement learning framework to the prediction problem of postoperative sepsis, fully utilizing the iterative feedback ideas and reward mechanisms, and proposed an improved approach with Hilbert-Schmidt Independence Criterion (HSIC) (Gretton et al., 2007). Then, experimental verification was conducted on a dataset of real patient cases. Finally, the model was explained post hoc based on integrated gradients algorithm. One of our novelties lies in the integration of HSIC into the Actor-Critic framework of RL to measure the independence between intraoperative temporal vital signs and its statistics. The main contributions of our work include the following four points:

(1) Based on the Actor-Critic framework of reinforcement learning, we proposed an interpretable sepsis prediction model, which takes feature selection as an action and evaluates it based on the accuracy of the prediction results. We employed the Actor model for feature selection and the Critic model for evaluation. Meanwhile, this is also one of our innovative points.
(2) In the proposed approach, we selected features just from preoperative static features and intraoperative statistical features, but can achieve similar effect as using all static, statistical, and intraoperative temporal monitoring signs. This is another novelty of this paper.
(3) The proposed model is visually explained and analyzed using the integrated gradient algorithm.
(4) Finally, we experimentally verified the model proposed in this paper based on real medical patient cases. The experimental results can well explain the prediction results and have a good auxiliary effect on clinical doctors.

## 2 Related works

### 2.1 AI for sepsis prediction

Numerous research teams have been seeking to use AI methods to identify patients with early symptoms of sepsis, including Logistic Regression (LR), Elastic-Net penalized Logistic Regression (ENLR), Support Vector Machine (SVM), Naïve Bayes (NB), K-Nearest Neighbors (KNN), Random Forest (RF), Adaptive Boosting Classifier (AdaBoost), Gradient Boosting Decision Tree (GBDT), eXtreme Gradient Boosting (XGBoost), Multi-Layer Perceptron (MLP), Neural Network (NN), Recurrent Neural Network (RNN), Long Short-Term Memory (LSTM), Temporal Convolutional Network (TCN), and so on.

Combined natural language processing (NLP) analysis of clinical notes with structured electronic medical record (EMR) data, a Sepsis Early Risk Assessment (SERA) algorithm was developed by Goh et al. (2021) to improve sepsis risk prediction. Compared to clinical doctors, the AI algorithm model can increase the early detection rate of sepsis by 32% and has a high prediction accuracy (AUC 94%, sensitivity 87%, specificity 87%) within 12 h before patient diagnosis, providing a golden window for clinical treatment.

Chen et al. (2023) conducted a retrospective analysis of 677 patients received liver transplant (LT) from January 2015 to January 2020 was retrospectively extracted from the big data platform of Third Affiliated Hospital of Sun Yat-sen University. They selected 8 features through Lasso regression to construct machine learning models and analyzed their importance. Seven predictive models were constructed for postoperative sepsis within 7 days in LT recipients using machine learning (ML) technology, including LR, SVM, RF, GBDT, AdaBoost, Gaussian Naive Bayes (NB), and MLP. The experimental results indicate that the overall performance of random forest is the best, with an accuracy of 71.6%, sensitivity of 62.1%, and specificity of 76.1%. However, there was no interpretability analysis.

In a different clinical context, another multicenter retrospective study by Liu et al. (2025) developed and validated an explainable prediction model for sepsis in patients with intracerebral hemorrhage. They evaluated nine machine learning algorithms and found that the Categorical Boosting (CatBoost) model achieved the best discriminative performance. Importantly, they employed SHapley Additive exPlanations (SHAP) to interpret the final model, providing insights into feature contributions, an aspect that remains underexplored in many postoperative sepsis prediction studies.

Li and Wang (2023) proposed a prediction model (TCASP) for the incidence of sepsis infection in ICU patients, by combining TCN and attention mechanism. The model was verified on the Medical Information Mart for Intensive Care III. In terms of area under the receiver operating characteristic curve (AUROC) and area under the precision-recall curve (AUPRC), the proposed model has improved by 6.4% and 3.9% respectively compared to Logistic regression, Insight, RNN, LSTM, and TCN. Similarly, this method remains a black box model, only improving the performance of the model.

Based on the vital signs and laboratory tests, Wu et al. (2024) developed an accurate and robust method for early detection of sepsis with an ad-hoc alarm reduction in ICUs by integrating customized down-sampling process, dynamic sliding window and XGBoost. The method can offer well-performed sepsis prediction approximate 5 h before onset. The proposed method achieved the highest AUC score on PhysioNet datasets, compared with Naïve Bayes, SVM, RF and Artificial Neural Network (ANN).

Based on time-series data, Tang et al. (2024) proposed predictive models for early sepsis prediction, utilizing both CNN-Transformer and LSTM-Transformer architectures. Compared with traditional recursive neural networks, the proposed model shows significant improvement in performance. In addition, the authors used the SHAP algorithm to visualize the weight distribution of different features, which enhanced the interpretability of the model.

Focusing specifically on postoperative settings, Bhambhvani et al. (2025) applied machine learning to predict sepsis after endourologic kidney stone surgery. They constructed five supervised learning models, including elastic net penalized logistic regression, random forest, neural network, support vector machine, and naive Bayes. Their results identified random forest as the optimal model, though the study did not include interpretability analysis.

For the aforementioned literatures, we have distilled a comprehensive summary focusing on two aspects: algorithmic information and interpretability, as shown in Table 1.

**Table 1:** Literatures of AI for sepsis prediction.

No.	Year	Authors	Algorithms info.	Interpretability
1	2021	Goh et al. (2021)	SERA	NO
2	2023	Chen et al. (2023)	LR, SVM, RF, GBDT, AdaBoost, NB, and MLP	NO
3	2023	Li and Wang (2023)	TCASP, LR, Insight, RNN, LSTM, and TCN	NO
4	2024	Wu et al. (2024)	XGBoost, NB, SVM, RF, ANN	NO
5	2024	Tang et al. (2024)	CNN-Transformer, LSTM-Transformer, RNN, LSTM	SHAP
6	2025	Liu et al. (2025)	Nine algorithms including CatBoost	SHAP
7	2025	Bhambhvani et al. (2025)	ENLR, RF, NN, SVM, NB	NO

### 2.2 RL for sepsis treatment

Reinforcement learning predominantly serves the purpose of intelligent auxiliary decision-making within the treatment of sepsis, rather than prediction.

A critical aspect of sepsis treatment is early fluid resuscitation, though optimal dosing remains controversial. To address this, Liang and Jia (2025) proposed a neural network-based reinforcement learning approach to estimate optimal multi-stage fluid resuscitation doses for septic patients. Their method was estimated to reduce average SOFA scores by 23.71%, demonstrating the potential of RL to optimize this critical yet controversial aspect of sepsis treatment.

Yu and Huang (2023) proposed a deep inverse reinforcement learning approach with mini-tree model to evaluate sepsis treatments, and the results show that the proposed methods can achieve more efficient treatment policies with higher reliability compared to those used by the clinicians.

Wu et al. (2023) developed a weighted dueling double deep Q-network with embedded human expertise in optimal treatment of sepsis, and achieved excellent performance.

Regarding the decision-making problem of heparin dosage in sepsis patients, Liu et al. (2024) proposed a model called SOFA-MDP using SOFA score as the state of Markov decision process, which provides instructive support for clinical decision-making.

Beyond treatment efficacy, Wang S. et al. (2025) incorporated differential privacy into deep reinforcement learning for sepsis treatment, enabling privacy-preserved clinical decision-making while mitigating pulmonary complications associated with sepsis.

### 2.3 Interpretability in medical field

The interpretability in medical field is mainly divided into post-hoc interpretability analysis and ad-hoc interpretable modeling.

Post-hoc interpretability analysis primarily consists of feature analysis, saliency, proxy, explaining-by-case and explaining-by-Text. Van Molle et al. made decisions on skin lesion classification through visual convolutional neural networks (Van Molle et al., 2018). Zech et al. (2018) developed a deep learning model for chest radiography and conducted interpretability analysis using class activation mapping (CAM) (Zhou et al., 2016). They reported the risk that deep learning models may make incorrect decisions by capturing features unrelated to the disease, such as metal markers. Ardila et al. (2019) proposed a deep learning algorithm to predict lung cancer risk and used the integral gradient (Sundararajan et al., 2017) method to derive saliency maps for explanation. Che et al. (2017) applied knowledge distillation to deep model, which has good interpretability in the context of electronic health record prediction. Pereira et al. (2018) studied the interpretability of automatically extracted features and applied it to brain lesion segmentation. Mutual information was used for global interpretation, while a variant of LIME (Ribeiro et al., 2016) was casted for local interpretation. Li et al. (2022) developed an interpretable model to predict the risk of death in patients with sepsis in the intensive care unit (ICU) and explored prognostic factors for sepsis using SHapley Additive explanation (Lundberg and Lee, 2017) method. Similarly, Wang Y. et al. (2025) developed an interpretable early mortality prediction model for sepsis using four machine learning algorithms on the MIMIC-IV database, with external validation in a Chinese teaching hospital. XGBoost achieved the best performance (AUROC 0.873), and SHAP was used to provide interpretable mortality predictions. Extending the application of interpretable ML to other clinical domains, Zuo and Yang (2025) employed XGBoost with SHAP to assess depression risk in stroke patients, and developed a web-based calculator to facilitate clinical use. Based on counterfactual inference, Xie et al. (2023) designed an interpretable sepsis prediction model and SHAP was used to rank the global and local feature importance.

Ad-hoc interpretable modeling mainly includes interpretable representation and model renovation. Fang and Yan (2020) designed a pyramid input-pyramid output feature abstraction network (PIPO-FAN) with multiple arms for multi-organ segmentation. Biffi et al. (2018) constructed a model for sepsis classification and provided an explanation through activation maximization technique.

## 3 Proposed method

To date, most models for predicting postoperative sepsis suffer from limited interpretability, which undermines their clinical credibility. To address this gap, this paper proposes a reinforcement learning-guided interpretable framework for postoperative sepsis prediction. Our objective is to develop a method that, using only preoperative static features and intraoperative statistical features, achieves predictive performance comparable to that of a baseline model that additionally incorporates intraoperative temporal monitoring signs.

Postoperative sepsis prediction is typically framed as a binary classification task within supervised learning. Feature selection is a critical step in this process, as it directly influences both model accuracy and interpretability. Conventional approaches select features based on criteria such as global importance scores. However, in clinical practice, the relevance of features can vary considerably across individual patients, exhibiting strong heterogeneity. Reinforcement learning, particularly through the Actor-Critic framework, offers a promising alternative by iteratively improving decision-making through value-based evaluation, which inherently supports interpretability. In this study, we treat feature selection as a sequential decision process: the Actor model is responsible for selecting features, while the Critic model evaluates these choices based on the accuracy of the resulting predictions. By integrating the strengths of both components, we aim to construct an interpretable and precise predictive model tailored to postoperative sepsis prediction.

### 3.1 Overall framework

**Figure 1:**
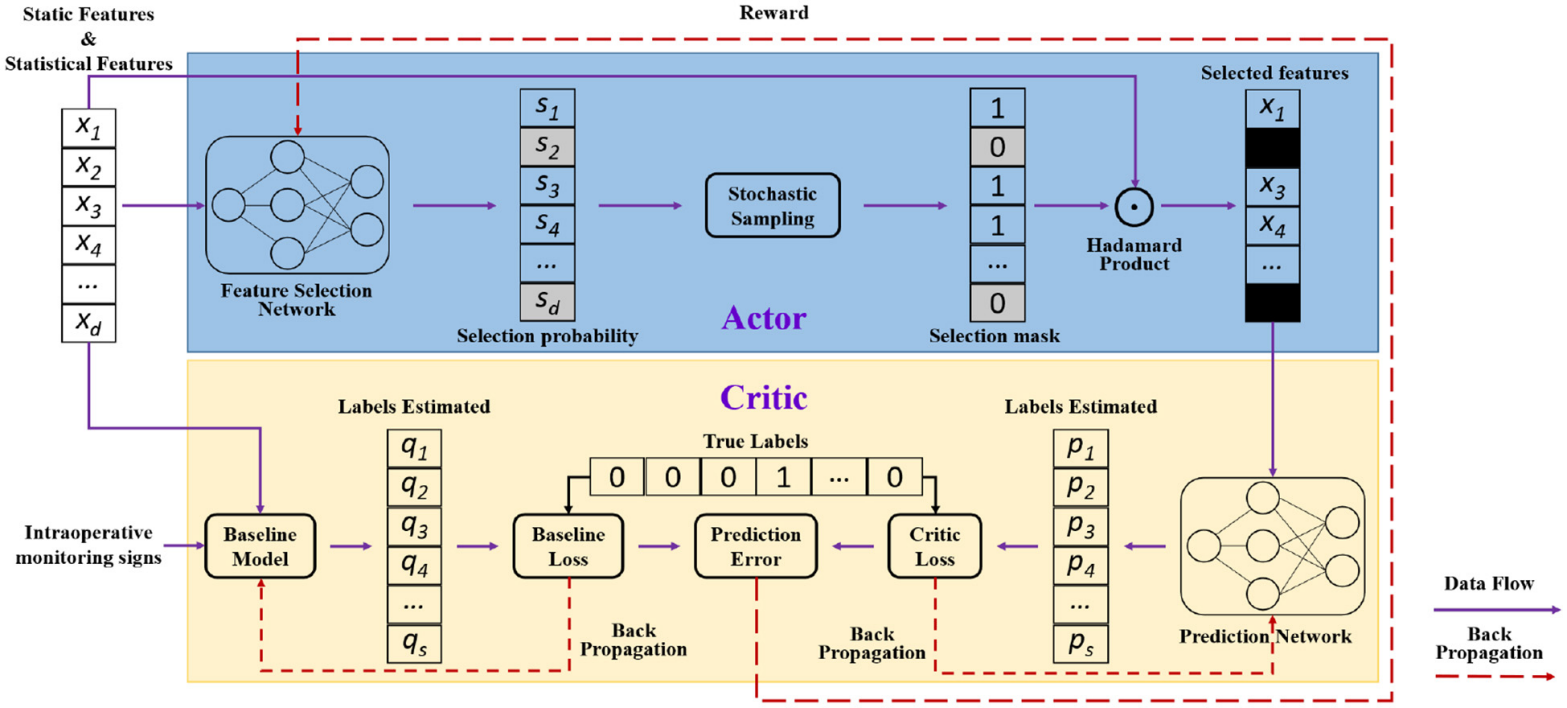
Overall framework of our method.

Based on the static, statistical and temporal features of patients, we constructed a prediction model iteratively with the Actor-Critic framework of reinforcement learning. And the overall framework of our approach is illustrated in Figure 1. As a fusion of value-based (e.g., Q-learning) and policy-based (e.g., policy gradient) algorithms, the Actor-Critic structure includes two parts: Actor model and Critic model. In our proposed approach, the Actor model is tasked with feature selection, with the aim of selecting the optimal subset of features for the prediction network. While the Critic model is dedicated to postoperative sepsis prediction, with the goal of accurately predicting whether the patient will develop postoperative sepsis as much as possible. The Actor model consists of three components: the feature selection network, the stochastic sampling module, and the Hadamard product module. The Critic model, in turn, encompasses the prediction network and an additional baseline model constructed using cross-entropy loss and HSIC loss, which serves as a reference.

Firstly, data is input into the feature selection network to obtain the selection probability of each feature. Then, according to these probabilities, the input vector is sampled to form a selection mask, which is used to determine which features are ultimately selected. Finally, the prediction network receives the selected features as input and performs predictions. Both of the two networks are backpropagation trained based on true labels. The total loss of the Actor-Critic structure, which includes the Actor loss, Critic loss and Baseline loss, is calculated for updating the network parameters. On one hand, using the baseline model as a benchmark, the goal of the Critic prediction network, through reinforcement learning training, is to get the outcomes matching to those of the baseline model. On the other hand, the baseline network uses all features, including static, statistical, and temporal features (i.e. monitoring signs), while the Actor network only uses static and statistical features without including original temporal monitoring data features. Our Actor-Critic structure is capable of attaining comparable effect to the model utilizing all features, but with a smaller number of features. Therefore, the selected features are the main features, which have a certain degree of interpretability.

The changes in temporal monitoring data such as heart rate, blood pressure, and oxygen saturation play an important role in postoperative sepsis prediction. Well known, the mean represents the central tendency of a set of data, while the standard deviation represents the degree to which the data deviates from the mean, i.e., the fluctuation of the data. The larger the standard deviation, the greater the degree to which the data deviate from the mean. Therefore, statistical data composed of mean and standard deviation can describe the overall changes in each vital sign data of patients. Patients are more likely to develop postoperative sepsis when his/her vital signs data fluctuate greatly. In our model, the mean value and standard deviation of each monitoring indicator are extracted as statistical features. The formulas for calculating the mean *x*_*M*_ and standard deviation σ are as follows:


$$\begin{array}{l} x_{M}=\frac{1}{N}\overset{N}{\underset{i=1}{\sum }}x_{i},\sigma =\sqrt{\frac{1}{N}\overset{N}{\underset{i=1}{\sum }}{\left(x_{i}-x_{M}\right)}^{2}} \end{array}$$ (1)

Within the framework of reinforcement learning, we take static and statistical features of patients as the environmental state, and feature selection behavior as the action. The parameters of Actor feature selection network are updated by measuring the loss difference between the prediction network and baseline model, i.e., the reward of Actor model, which is defined as:


$$\begin{array}{l} R=-\left(L_{prediction}-L_{baseline}\right) \end{array}$$ (2)

where *R* is reward, *L*_*prediction*_ is prediction network loss and *L*_*baseline*_ is baseline model loss. We will provide a detailed description of them in the subsequent contents.

Let *X*_*s*_ be preoperative static data, *X*_*m*_ be intraoperative statistical data, *X*_*d*_ = (*X*_*s*_, *X*_*m*_), and *X*_*v*_ be intraoperative temporal vital signs (i.e. monitoring signs). Next, we will depict each part of our approach.

### 3.2 Actor model for feature selection

Feature selection is completed by the Actor model. With the feature selection network *N*_*fs*_, we can obtain the selection probability *S*_*d*_ of each feature. In the stochastic sampling process, Bernoulli sampling is applied, and a selection mask *M* is formed. Finally, through the Hadamard product, denoted as ⊙, we got the ultimately selected features $X_{d}^{*}$. Denote the stochastic sampling process as *Sampling*_*Bernoulli*_. Then, the whole feature selection process can be represented as follows:


$$\begin{array}{l} S_{d}=N_{fs}\left(X_{d}\right) \end{array}$$ (3)

$$\begin{array}{l} M=Sampling_{Bernoulli}\left(S_{d}\right) \end{array}$$ (4)

$$\begin{array}{l} X_{d}^{*}=X_{d}⊙M \end{array}$$ (5)

**Figure 2:**
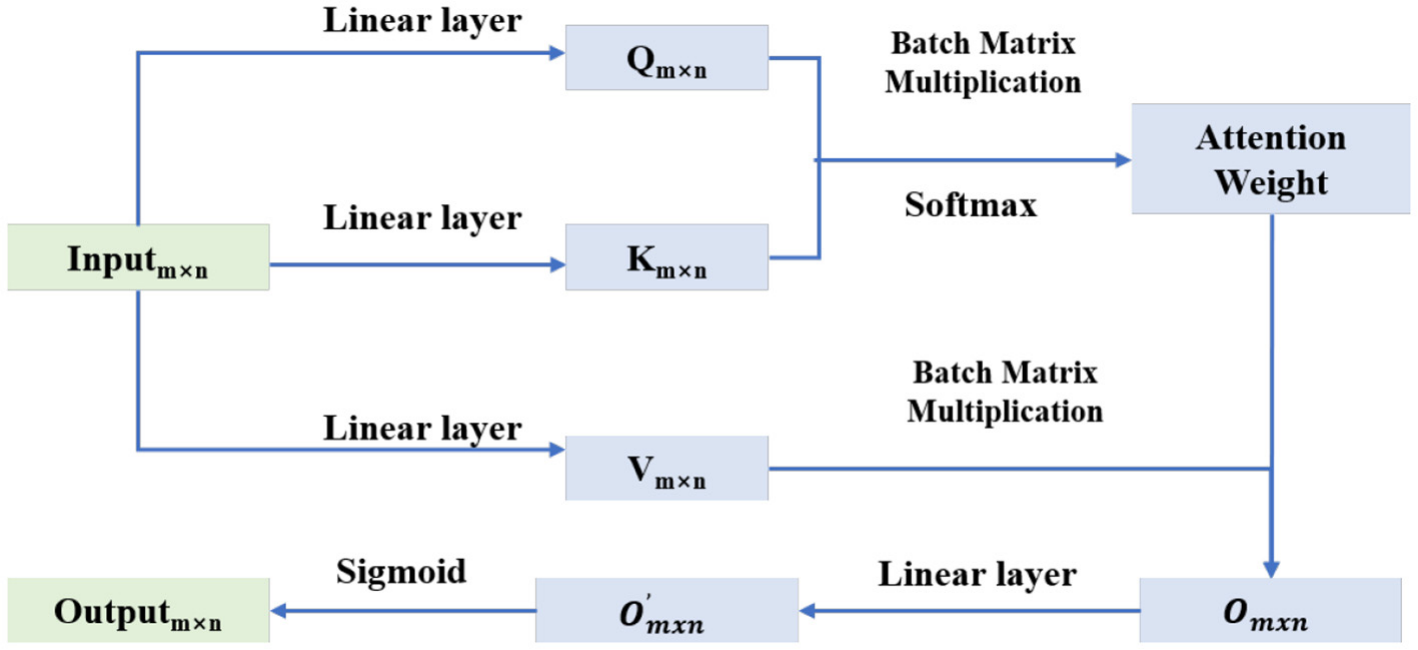
Structure of the feature selection network.

The essence of feature selection is to select the optimal subset of features for model construction. Similar to the way humans process information, the self-attention mechanism can focus the model's attention on important features, which is precisely the goal of feature selection. Therefore, in this paper, we employ a simple self-attention network as the feature selection network *N*_*fs*_ within the Actor model to complete the feature selection action. The schematic diagram of this simple self-attention network is provided in Figure 2.

The Self-Attention Mechanism is a technique that allows models to focus on information at different positions within a sequence when processing sequential data. Self-Attention Networks are a type of deep learning architecture that utilizes the self-attention mechanism to handle sequential data. These networks are capable of capturing long-range dependencies within sequences, making the model more effective when dealing with natural language or time series data. The core of a self-attention network is the self-attention layer, where each element of the sequence generates query (Query), key (Key), and value (Value) vectors. By calculating the similarity between the query and all keys, the model can determine which elements in the sequence are most important for the current element. Then, this information is used to weight and sum the corresponding value vectors to produce the output.

### 3.3 Prediction network

The prediction network of the Critic model is denoted as *N*_*prediction*_. It takes the finally selected feature $X_{d}^{*}$ as input and provides estimated labels which indicates the risk of postoperative sepsis. Let *s* be the sample size, and $p={\left(p_{1},p_{2},\cdots \;,p_{s}\right)}^{T}$ be the labels estimated. Then, we have


$$\begin{array}{l} p=N_{prediction}\left(X_{d}^{*}\right) \end{array}$$ (6)

**Figure 3:**
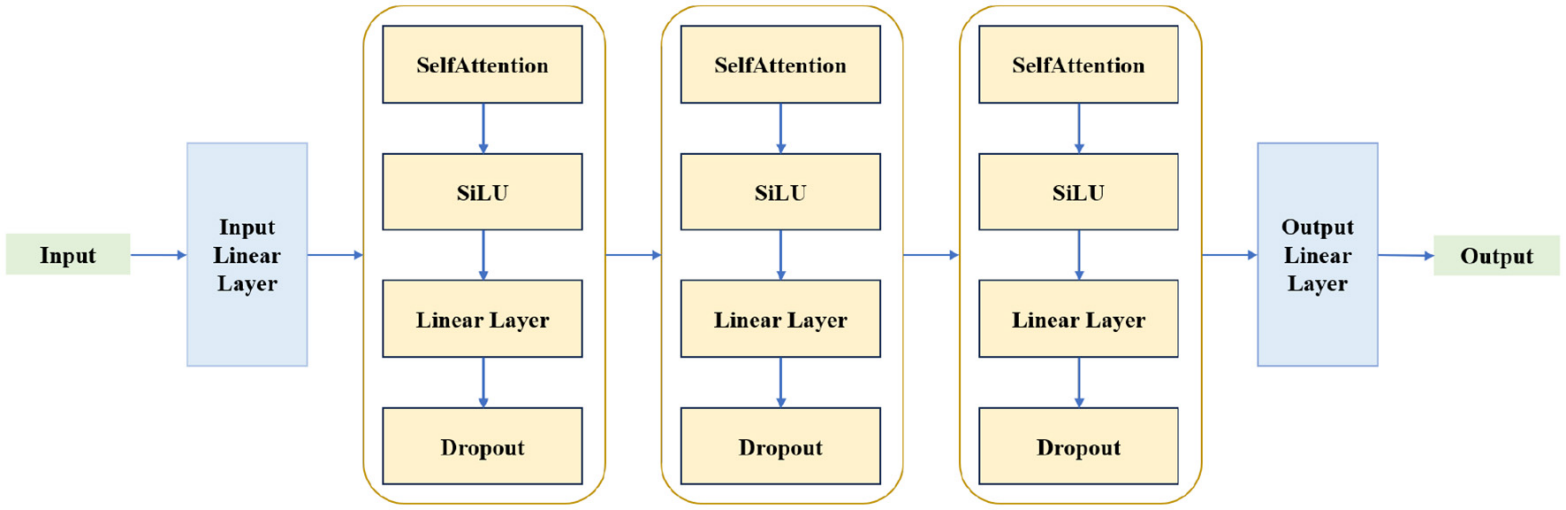
Structure of the prediction network.

We still employ the attention mechanism to construct the prediction network, which is a complex self-attention network, and its network structure is shown in Figure 3. The prediction network is composed of an input linear layer, three self-attention network layers, and an output linear layer. Each self-attention network layer includes a simple self-attention network (the same as the feature selection network), a SiLU layer, a linear layer, and a Dropout regularization layer.

### 3.4 Baseline model with TCN and HSIC

Patient's temporal monitoring data contains rich information. Statistical features can only reflect the overall trend of data, but cannot reveal the temporal information of data changes. We need to explore more useful features from monitoring data besides the mean and standard deviation. Temporal convolutional network (TCN) is just a good choice. In 2018, Bai S et al. applied convolutional neural network (CNN) to sequence data analysis tasks and proposed TCN (Bai et al., 2018). They found that TCN has strong perception ability for temporal data and can achieve excellent performance. TCN can avoid some shortcomings of recurrent neural network (RNN), such as gradient explosion and gradient vanishing. In addition, TCN has faster computational speed compared to RNN. TCN can extract temporal features from monitoring data and fully consider changes in vital signs. In this paper, we extract temporal features from monitoring data through a TCN as part of the input of the baseline model.

We not only extracted statistical features (i.e., mean and variance) from monitoring data, but also derived more temporal features from it by TCN. However, is there information redundancy between the extracted statistical and temporal features? Can we maximize the independence between the two kinds of features? This is an important issue worth considering. Therefore, we employed Hilbert-Schmidt Independence Criterion (HSIC) (Gretton et al., 2007) to measure the independence of statistical and temporal features, hoping to extract as much useful information as possible from monitoring data. HSIC is mainly used to measure the distribution differences between two variables, which is constructed based on covariance.

**Figure 4:**
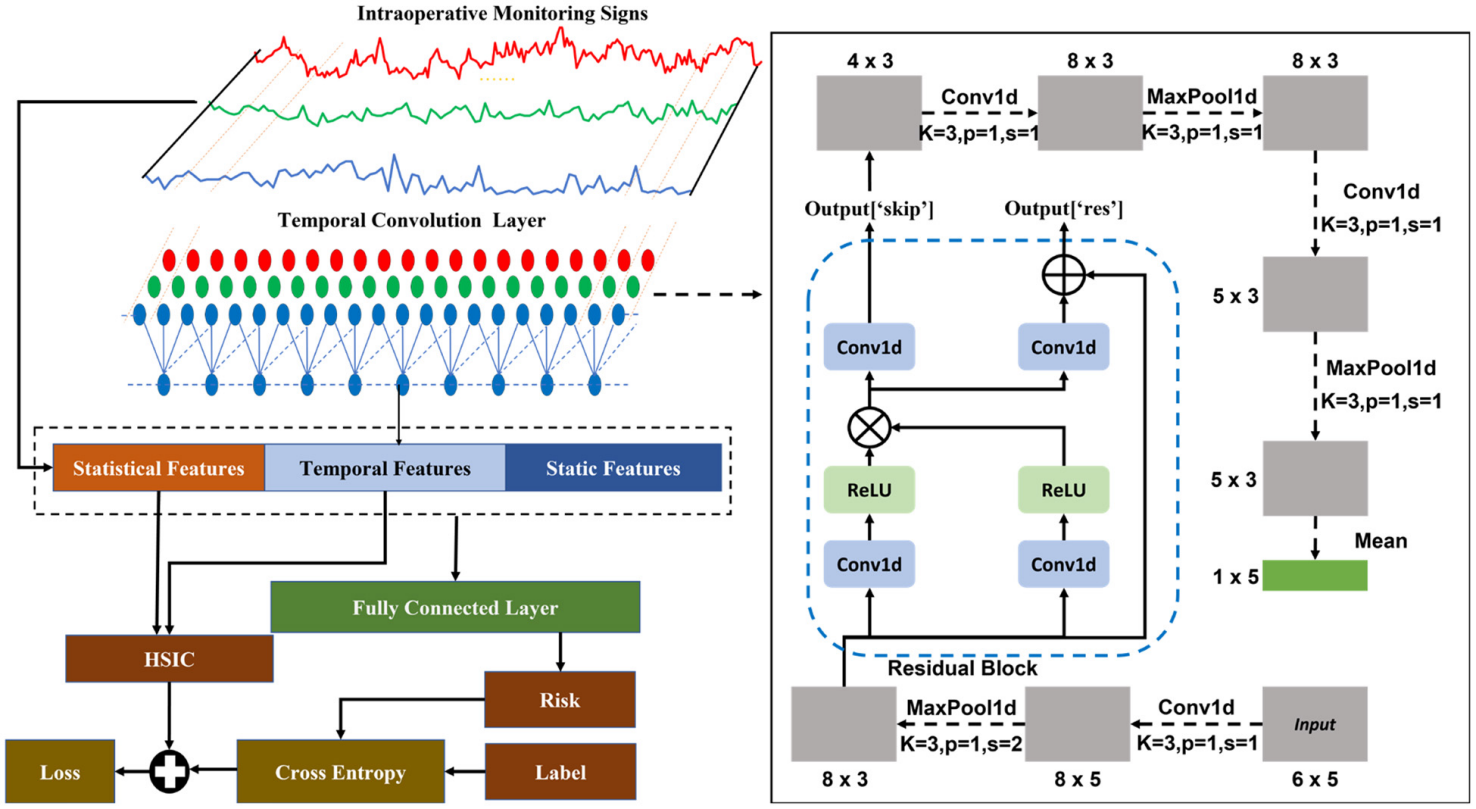
Structure of baseline model.

In our work, we constructed a baseline model as a benchmark for the Critic prediction network. The baseline model consists of two parts: a TCN and a fully connected layer. The structure of the baseline model is depicted in Figure 4. In addition to the statistical features of vital signs data, we also used TCN to extract temporal features of monitoring vital signs. The input of the baseline model includes all of the static, statistical, and temporal features.

Firstly, temporal features are extracted from intraoperative monitoring data of patients through the TCN network which is illustrated in the right half of Figure 4 in detail. Specifically, in the TCN, the vital signs data is convolved and pooled three times. At the same time, it is passed through a residual block. In the residual block, convolution and ReLU are the main operations that are performed on the input features. Meanwhile, we also extracted statistical features (mean and variance) from the monitoring data. Then, temporal and statistical features, along with static features of patients, are used as inputs to the fully connected layer to obtain an estimate of the patient's postoperative sepsis risk.

### 3.5 Loss function

Classification problems usually use cross-entropy loss function, which enables the model to converge faster. Cross-entropy is used to measure the difference between the two probability distributions obtained from the current training and the real distribution respectively. It characterizes the distance between the actual output (probability) and the expected output (probability). The smaller the value of the cross-entropy, the closer the two probability distributions are. However, when training the model, our main purpose is not purely for classification, but to hope that our approach can achieve an approximate effect with the baseline model. Therefore, based on cross entropy, we modified the loss functions.

The loss of baseline model consists of two parts: fully connected layer loss and HSIC loss. HSIC measured the independence between statistical and temporal features. The baseline loss *L*_*baseline*_ can be formulated as:


$$\begin{array}{l} L_{baseline}=L_{fcl}+L_{hsic} \end{array}$$ (7)

where *L*_*fcl*_ is a cross-entropy loss produced by the fully connected layer, and *L*_*hsic*_ is the HSIC loss which takes Gaussian kernel function as its Reproducing Kernel Hilbert Space (RKHS). Similar to mutual information, HSIC can be used to measure the independence between two variables. It is the Hilbert Schmidt norm of the cross-covariance operator between two distributions in a RKHS. The formulation of HSIC is:


$$\begin{array}{l} HSIC\left(P_{xy},F,G\right)=\|C_{xy}{\|}_{HS}^{2} \end{array}$$ (8)

where the norm ||▪||_*HS*_ is defined as:


$$\begin{array}{l} {\left\|A\right\|}_{HS}=\sqrt{\underset{i,j}{\sum }a_{i,j}} \end{array}$$ (9)

*F* is the RKHS of *X* with kernel function *k*(*x, x*′) = 〈ϕ(*x*), ϕ(*x*′)〉, *G* is the RKHS of *Y* with kernel function *l*(*x, x*′) = 〈φ(*x*), φ(*x*′)〉. *C*_*xy*_ is the cross-covariance operator which can be written as


$$\begin{array}{l} C_{xy}=E_{xy}\left[\left(\phi \left(x\right)-\mu_{x}\right)⊗\left(\varphi \left(y\right)-\mu_{y}\right)\right] \end{array}$$ (10)

where μ_*x*_ = *E*_*x*_ϕ(*x*), μ_*y*_ = *E*_*y*_φ(*y*), and ⊗ is the tensor product. With an appropriate kernel choice such as Gaussian kernel function


$$\begin{array}{l} k\left(x,y\right)=\exp\left(-\frac{{\left\|x-y\right\|}^{2}}{2\sigma^{2}}\right) \end{array}$$ (11)

HSIC is zero if and only if the random variables *X* and *Y* are independent (Ma et al., 2020).

The prediction network loss *L*_*prediction*_ is just the cross entropy between the estimated and true labels. Then, we set the loss of Critic model as:


$$\begin{array}{l} L_{critic}=L_{baseline}+L_{prediction}=L_{fcl}+L_{hsic}+L_{prediction} \end{array}$$ (12)

For the Actor model, the loss function is defined as:


$$\begin{array}{l} L_{actor}=RL_{fs}-\lambda \bar{S_{d}} \end{array}$$ (13)

where *R* is the reward of reinforcement learning mentioned before, *L*_*fs*_ is the loss of feature selection network which is defined as the cross-entropy between selection probability and stochastic sampling result, λ is the regularization coefficient, and $\bar{S_{d}}$ is mean of selection probability *S*_*d*_. According to the definition of *R*, *L*_*actor*_ can be reformulated as:


$$\begin{array}{l} L_{actor}=-\left(L_{prediction}-L_{baseline}\right)L_{fs}-\lambda \bar{S_{d}} \end{array}$$ (14)

and then,


$$\begin{array}{l} L_{actor}=-\left(L_{prediction}-L_{fcl}-L_{hsic}\right)L_{fs}-\lambda \bar{S_{d}} \end{array}$$ (15)

Finally, the total loss of our approach is set to be:


$$\begin{array}{l} L_{total}=L_{actor}+L_{critic}=L_{actor}+L_{fcl}+L_{hsic}+L_{prediction} \end{array}$$ (16)

s

It can also be rewritten as:


$$\begin{array}{r} L_{total}=-\left(L_{prediction}-L_{fcl}-L_{hsic}\right)L_{fs}-\lambda \bar{S_{d}}+L_{fcl}\\ +L_{hsic}+L_{prediction} \end{array}$$ (17)

## 4 Experiments and results

We conducted experiments on the dataset from Southwest Hospital in Chongqing, China, and compared the proposed approach with other state-of-the-art algorithms. We implement our model with PyTorch and conduct all our experiments on a server with Ubuntu 16.04 LTS operating system, Intel Xeon e5-2650 V4 processor and Nvidia GTX 1080 Ti GPU, with 64G memory.

### 4.1 Dataset description

The dataset employed in this experiment is collected from surgical patients at Southwest Hospital in Chongqing, China, spanning from 2014 to 2018. This study was approved by the Ethics Committee of the First Affiliated Hospital of Army Medical University, PLA, and the Approved No. of ethic committee is KY201936.

The total sample size of this dataset is 1,538, including 82 positive samples and 1,456 negative samples. We randomly divide the dataset into two parts: training set and testing set. The training set accounts for 80%, while the remaining 20% is the testing set.

The original patient data includes preoperative examination and demographic data, as well as intraoperative monitoring data. There are 20 indicators of preoperative static data, with specific attribute indicators shown in Table 2. And the intraoperative monitoring data information is illustrated in Table 3, including 5 indicators.

**Table 2:** Preoperative examination and demographic indicators.

No.	Indicator name	Abbreviation
1	5′-nucleotidase (5′-NT)-venous blood	5′-NT
2	Chlorine (Cl)-venous blood	Cl
3	Glomerular filtration rate (GFR)-venous blood	GFR
4	Alanine aminotransferase (ALT)-venous blood	GLT
5	Creatinine (Crea)-venous blood	Crea
6	Cholic acid (CG)-venous blood	CG
7	Potassium (K)-venous blood	K
8	Age	Age
9	Sodium (Na)-venous blood	Na
10	Transparent tube type-urine	TTT-U
11	α-L-fucosidase (AFU)-venous blood	AFU
12	Urea-venous blood	Urea
13	Fibrinogen (Fbg)-venous blood	Fbg
14	Aspartate aminotransferase (AST)-venous blood	AST
15	Cystatin C (Cys-C)-venous blood	Cys-C
16	Phosphorus (P)-venous blood	P
17	Fibrinogen degradation product (FDP)-venous blood	FDP
18	D-dimer-venous blood	D-Dimer
19	Osmotic pressure (Osm)-venous blood	Osm
20	Platelet count (PLT#)-venous blood	PLT#

**Table 3:** Intraoperative monitoring indicators.

No.	Indicator name	Abbreviation
1	Heart rate	HR
2	Systolic blood pressure	SBP
3	Diastolic blood pressure	DBP
4	Blood oxygen saturation	SPO2
5	Central venous pressure	CVP

### 4.2 Sepsis prediction experiments

In this paper, the postoperative prediction of sepsis is modeled and analyzed as a binary classification problem, and the performance of the model can be evaluated through the confusion matrix. According to the ground truth and prediction results, true positive (TP), true negative (TN), false positive (FP) and false negative (FN) can be calculated. Accuracy (ACC), F1-score (F1), Sensitivity, Specificity, Area Under Roc Curve (AUC), and Average Precision (AvgPrec) were used to evaluate the performance of our approach.

To verify the effectiveness of the model's predictive performance, we will compare the model proposed in this paper with other algorithms. Specific methods include commonly used models in the medical field: NB (Wu et al., 2024), KNN, MLP (Chen et al., 2023), RF (Chen et al., 2023), and XGBoost (Wu et al., 2024).

In the model validation process, the feature selection network of Actor is a self-attention network. In the feature selection network, each linear layer has an input and output feature count of 30, which is the sum of the number of static and statistical features. As mentioned above, the prediction network is a complex self-attention network with 30 input nodes and 64 hidden nodes in each layer. The dropout rate of the dropout layer is 0.5.

**Figure 5:**
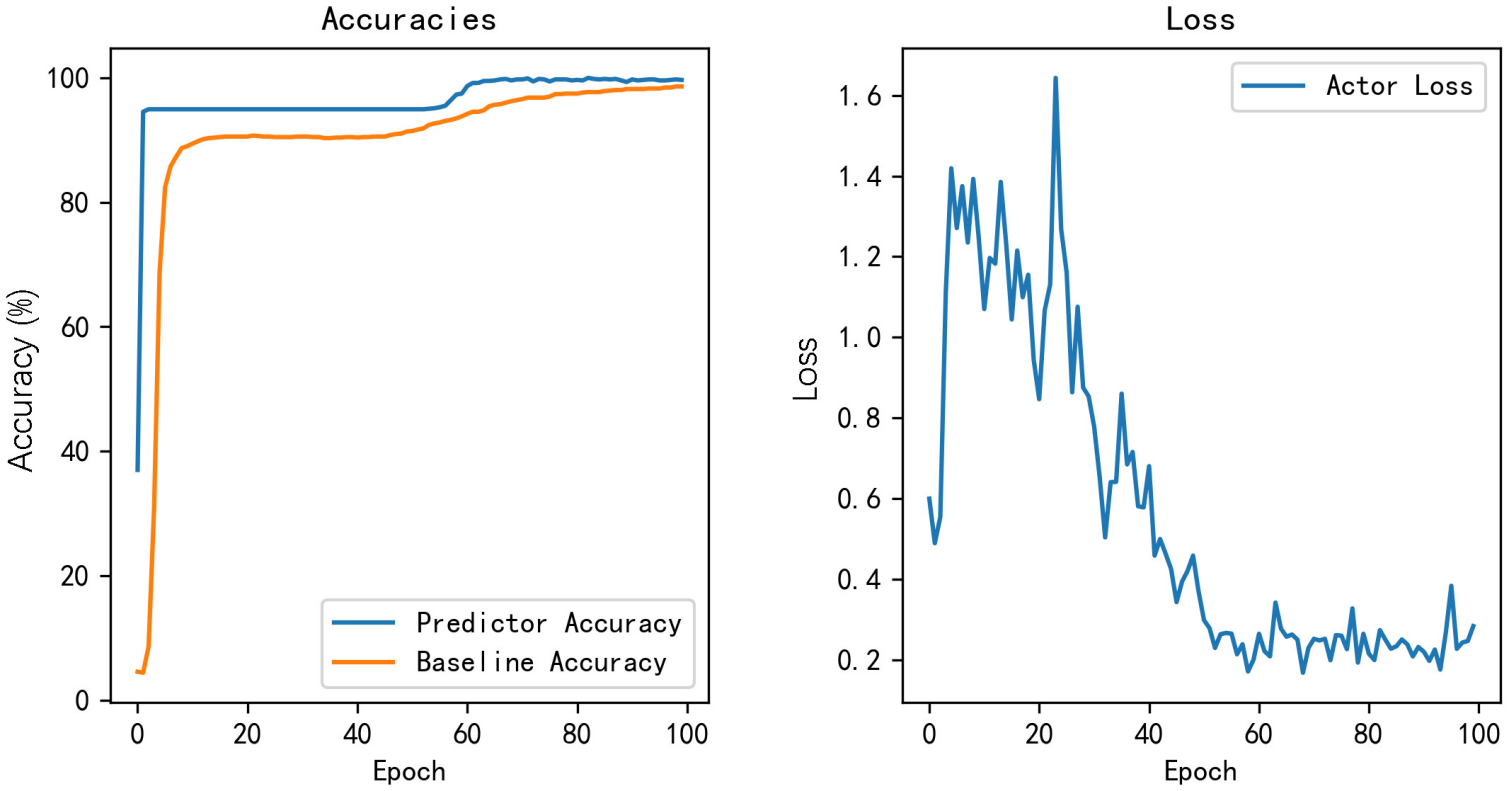
Accuracy and loss of our model.

The Baseline network is a 10-layer TCN network with residual networks for time series. The input includes the patient's static data, intraoperative time series monitoring data and statistical features. The HISC independent intraoperative time series data features and statistical features are used. The experiment is based on 100 epoch training using the backpropagation algorithm. The initial learning rate is set to 0.01 and adjusted dynamically by exponential learning in the later stage. Each batch size is set to 32. Based on the validation dataset, we calculate the prediction accuracy of the prediction network and baseline network and the overall loss function of the model. As shown in Figure 5, as the model was trained, the accuracy has converged after 60th epoch which indicated that the model has learned useful features for prediction. And the loss tends to be stable after training for 60 epochs also. Therefore, based on the prediction results of the baseline, we can adjust the parameters of the feature selection network (Actor) and prediction network in reverse to enable the model to better select effective feature indicators.

**Figure 6:**
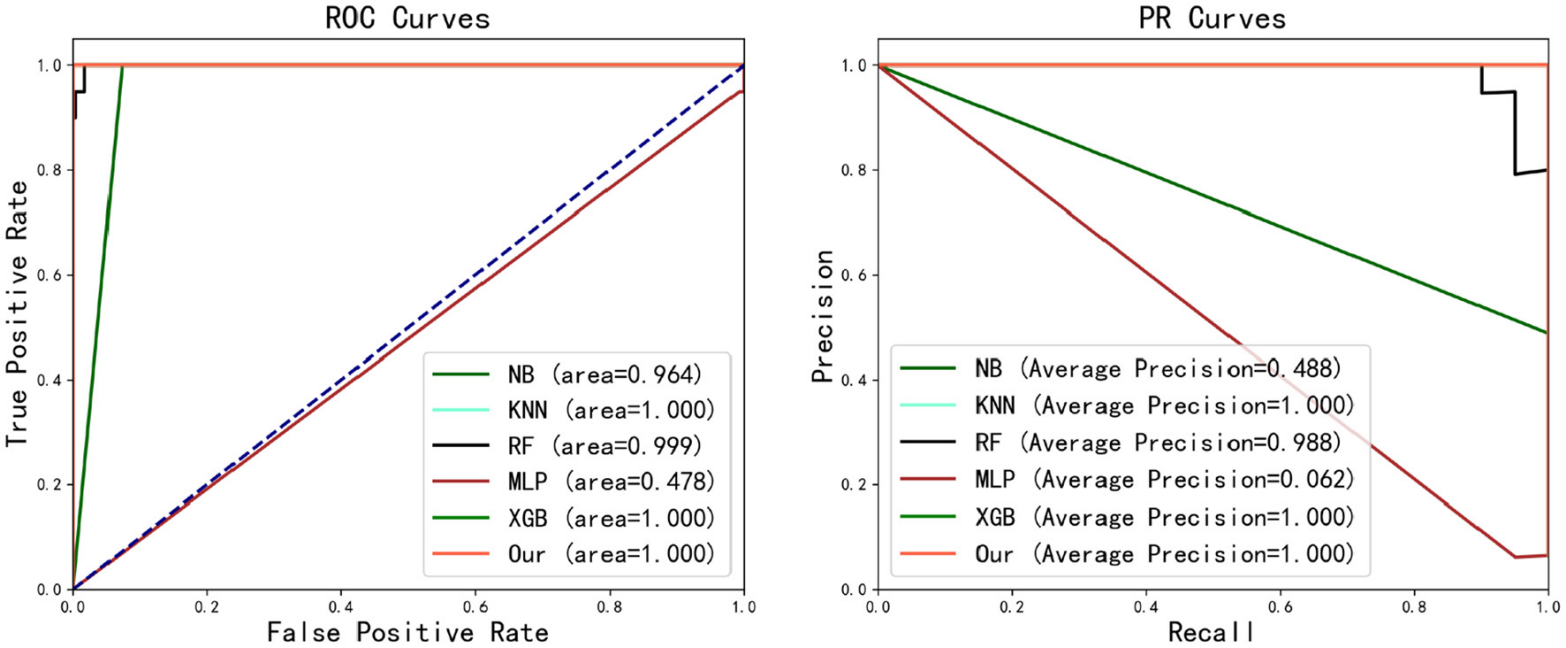
ROC and PR curve of various methods.

From the experimental results shown in Table 4, it can be seen that the dataset used has good performance in terms of separability on various models, except for MLP. It is evident that the proposed method outperforms all the comparative algorithms. On the test dataset, the proposed method is able to accurately predict whether a patient's case will develop postoperative sepsis. KNN and RF have achieved relatively good predictive results, although there are certain cases of missed diagnoses. XGBoost also performs well, but there is a certain degree of misdiagnosis, predicting 2 (288 in total) negative samples as positive. The NB model is similar to XGB, but it has a more severe misdiagnosis situation, incorrectly classifying 21 negative samples as positive. The MLP model performed the poorest, incorrectly predicting all samples to be negative. In terms of AUC and average precision, our method, XGB, and KNN are the best. The ROC and PR curve results are shown in Figure 6. It can be seen from the figure that our proposed method is the best. In the aspect of F1 score, our approach outperforms the others, while XGB and KNN also demonstrate good results. The proposed approach, along with XGB and NB, exhibits perfect sensitivity. The specificity of all methods is quite satisfactory.

**Table 4:** Experimental results.

Method	AUC	AvgPrec	ACC	F1	Sensitivity	Specificity
NB	0.964	0.488	0.932	0.656	1.000	0.927
KNN	1.000	1.000	0.994	0.947	0.900	1.000
MLP	0.478	0.062	0.935	0.000	0.000	1.000
RF	0.999	0.988	0.990	0.919	0.850	1.000
XGBoost	1.000	1.000	0.994	0.952	1.000	0.993
**Ours**	**1.000**	**1.000**	**1.000**	**1.000**	**1.000**	**1.000**

Bold values represent the best performance metrics.

**Figure 7:**
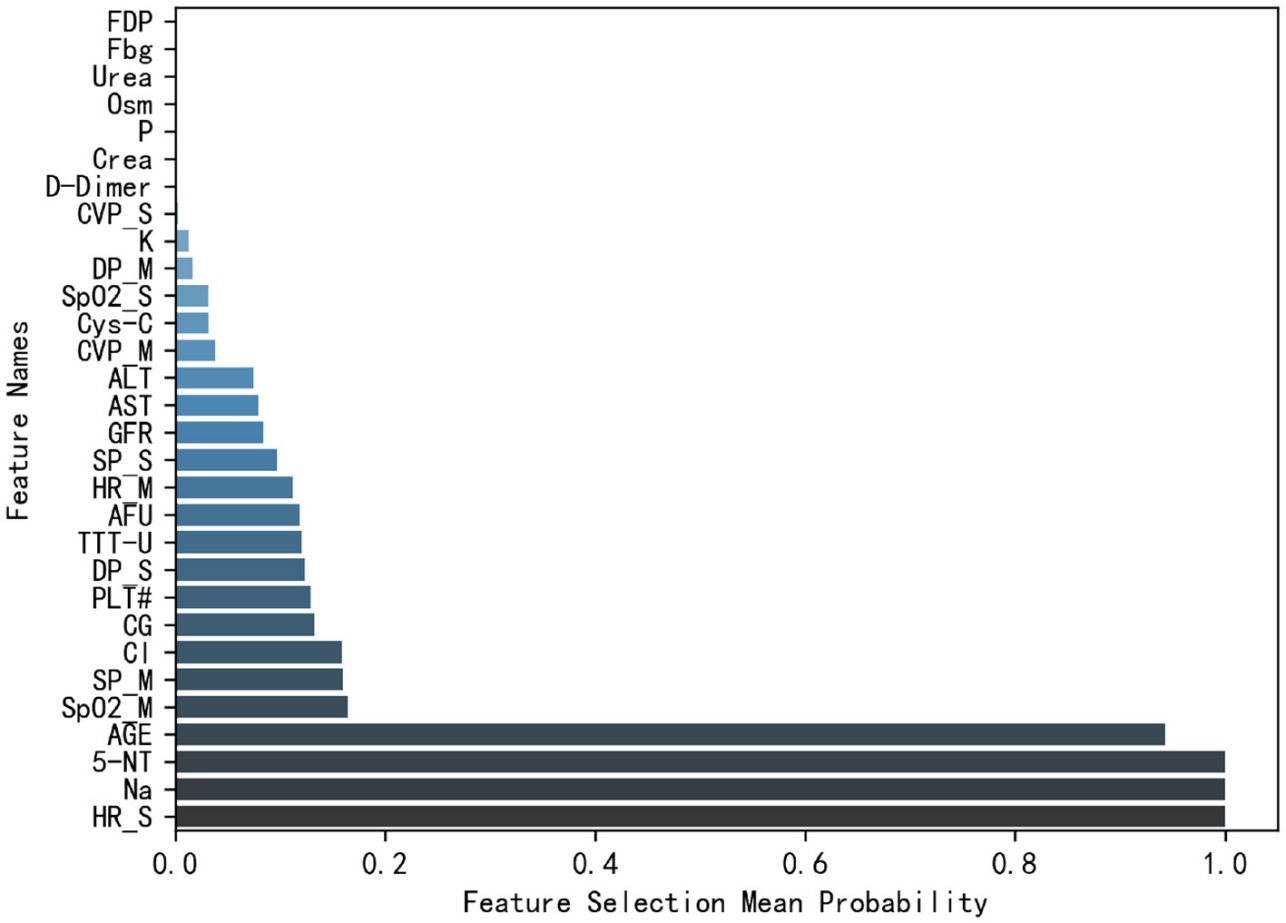
Feature selection probabilities ranking.

Also, we present the ranking of average feature selection probabilities in Figure 7. It can be observed that the most frequently used features include standard deviation of heart rate (HR_S), Na, 5-NT, and AGE.

### 4.3 Ablation experiments

To further validate the effectiveness of the proposed model, we conducted ablation analysis by replacing the feature selection network and the prediction network. Designate the simple self-attention network shown in Figure 2 and the complex self-attention network depicted in Figure 3 as SAN and CAN respectively. In the ablation experiment, the number of hidden layer nodes in the MLP is set to 100. The performance comparison of ablation experiments is as shown in Table 5. “SAN + CAN” indicates that the feature selection network employs SAN, while the prediction network uses CAN. Other combinations follow suit accordingly.

**Table 5:** Performance comparison of ablation experiment.

Method	AUC	AvgPrec	ACC	F1	Sensitivity	Specificity
CAN + CAN	0.970	0.564	0.935	0.000	0.000	1.000
CAN + MLP	0.954	0.572	0.938	0.095	0.050	1.000
SAN + MLP	0.944	0.880	0.981	0.833	0.750	0.997
MLP + CAN	0.986	0.821	0.955	0.500	0.350	0.997
MLP + MLP	0.949	0.946	0.994	0.950	0.950	0.997
**SAN** **+** **CAN**	**1.000**	**1.000**	**1.000**	**1.000**	**1.000**	**1.000**

Bold values represent the best performance metrics.

It is evident that our model, the combination of SAN and CAN, is optimal, with performance significantly better than other combinations. By comparing the results of SAN + MLP with our model and comparing MLP + CAN with MLP + MLP, it can be observed that simply replacing either the feature selection network or the prediction network may not easily achieve the desired effect. From Table 5, we can also learn that CAN + CAN and CAN + MLP clearly categorized almost all sample cases as negative, even when the samples were actually positive. In contrast, our model is capable of effectively distinguishing between negative and positive samples.

## 5 Interpretability cases

We visualize and explain the prediction results in two ways. First, the integrated gradient algorithm is used to visualize the attribution of the patient's input indicators through gradient visualization. Then, through the selection network of the model framework, individualized instance selection is used to explain the prediction results of the model by selecting corresponding features.

The Integrated Gradients algorithm combines the design ideas of direct gradient and divide-and-conquer of attribution techniques based on backpropagation such as DeepLift and LRP, satisfying the axioms of sensitivity and implementation invariance. Let the input be *x*, the baseline value be *x*′, and the function mapping be represented as *f*. The integral gradient for the *i*-th dimension of the input can be expressed as follows:


$$\begin{array}{l} \phi_{i}^{IG}\left(f,x,x^{\prime }\right)=\left(x_{i}-{x^{\prime }}_{i}\right)\int_{0}^{1}\frac{\delta f\left(x^{\prime }+\alpha \left(x-x^{\prime }\right)\right)}{\delta x_{i}}d\alpha \end{array}$$ (18)

Through the gradient method of integration, the patient's intraoperative monitoring data, preoperative examination data and intraoperative monitoring statistical data can be visualized. Here, we provide two example samples as follows. One is positive, and the other is negative.

**Figure 8:**
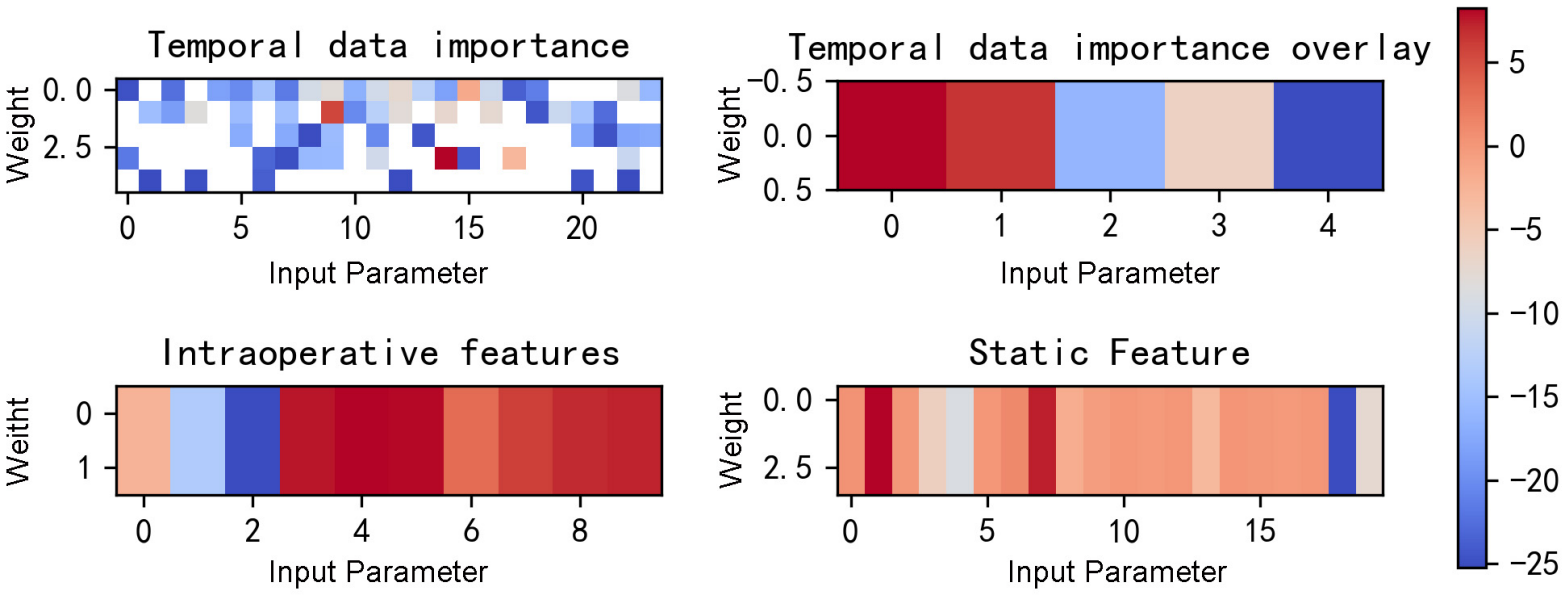
Gradient heat map of input data for the example negative patient.

**Figure 9:**
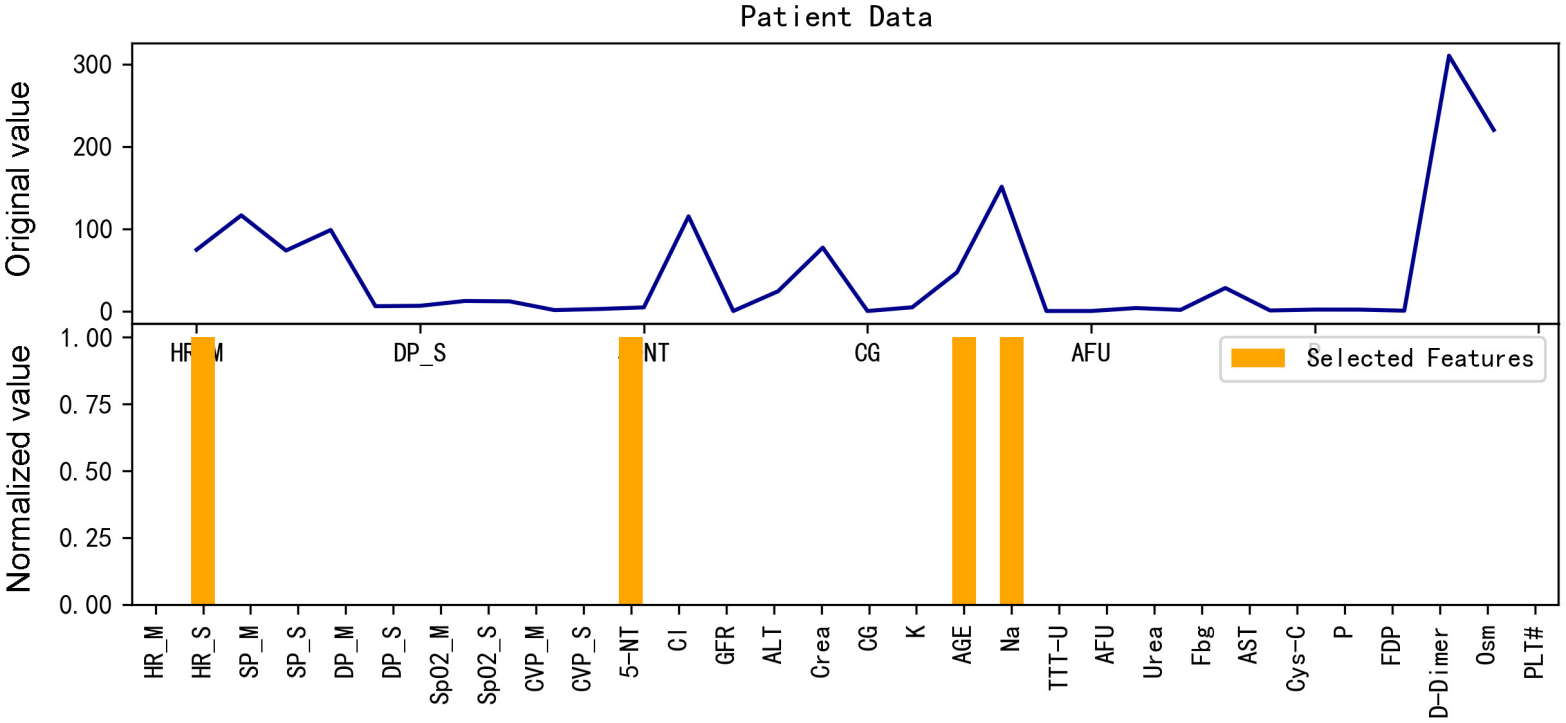
Input data and features selected for the example negative patient.

From Figures 8, 9, it can be seen that when our model predicts a sample as negative, it mainly focuses on a few features. For example, for the aforementioned negative sample, the model's weights are primarily concentrated on four features: HR_S, 5-NT, AGE, and Na.

**Figure 10:**
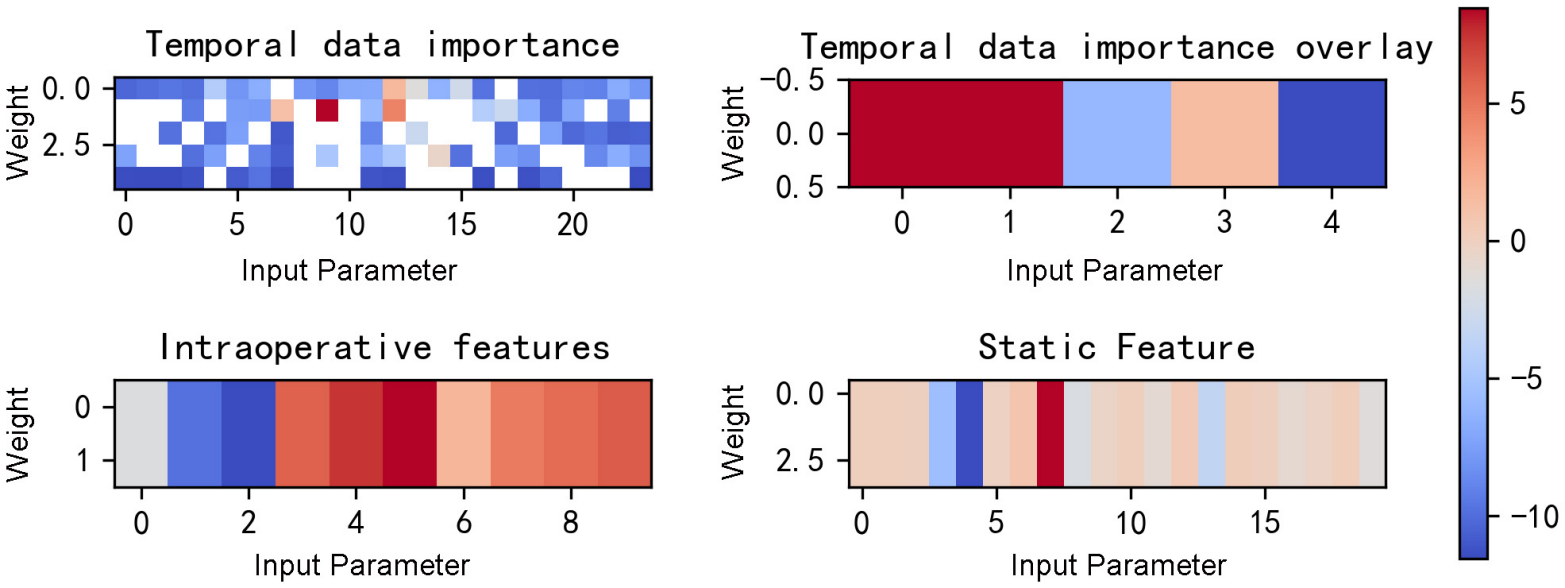
Gradient heat map of input data for the example positive patient.

**Figure 11:**
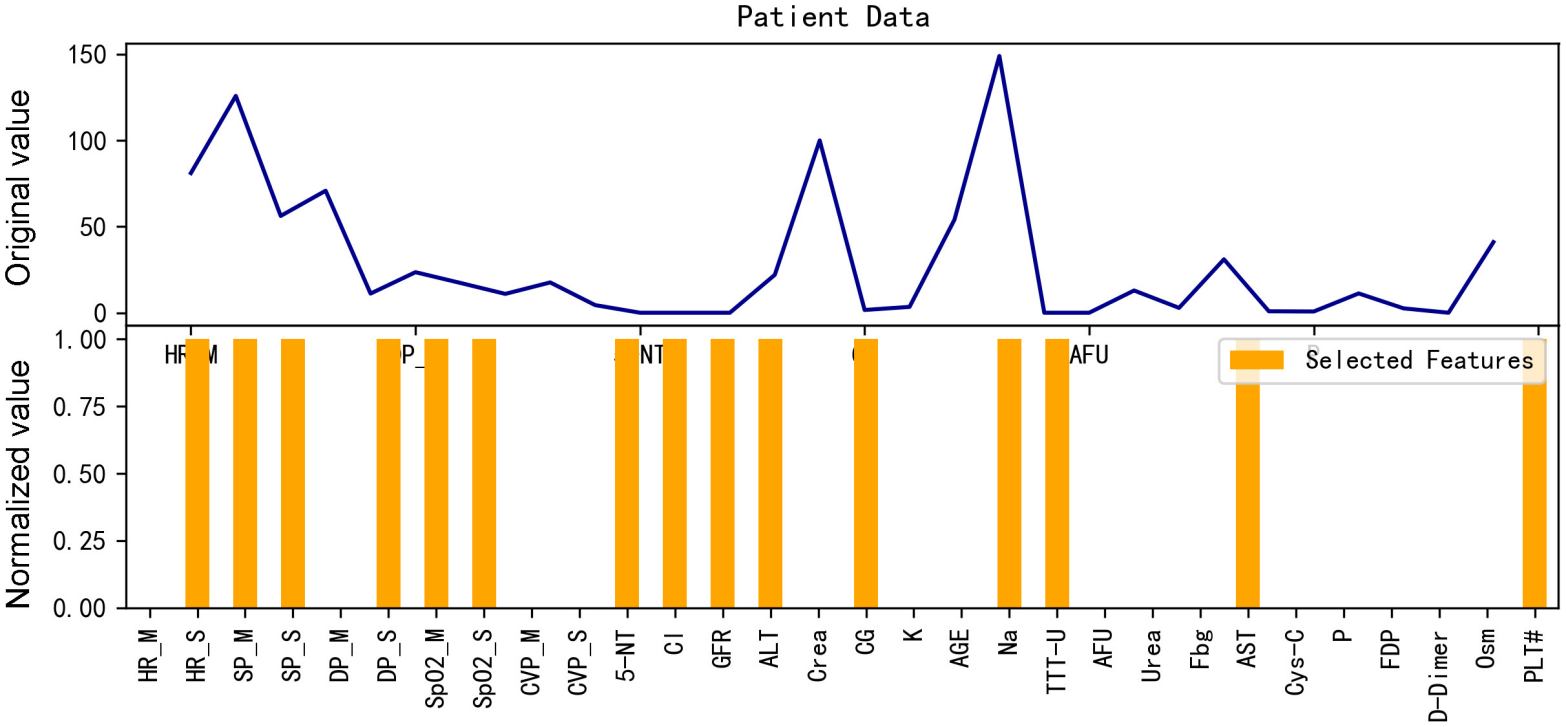
Input data and features selected for the example positive patient.

According to Figures 10, 11, we can deduce that our model focuses on a much larger number of features when predicting a sample as positive. Moreover, these features are quite dispersed, encompassing both preoperative features and intraoperative temporal features.

Comparing Figures 9, 11, we know that the model focuses on different features for negative and positive samples, which indicated that the model has learned the distinguishing features of patients. Through the Actor model, feature selection can be performed for each individual patient.

In the interpretability cases described above, the Actor network generates stochastic feature masks for each patient to select subsets of preoperative and intraoperative variables. These masks inherently reflect the relative importance of different clinical features for each specific prediction outcome. By analyzing the masks corresponding to these cases, we can identify which variables exert the greatest influence on the model's decision-making process for that particular instance. This direct linkage demonstrates that our method not only achieves competitive predictive performance with fewer features but also provides clinically meaningful, patient-level explanations, thereby enhancing the interpretability and trustworthiness of the model's predictions.

## 6 Discussion

Compared to previous methods, our study has three innovative points. Firstly, we took feature selection as an action of RL and evaluated it based on the accuracy of the prediction results. We employed the Actor model for feature selection and the Critic model for evaluation. Secondly, we selected features just from preoperative static features and intraoperative statistical features, but can achieve similar effect as using all static, statistical, and intraoperative temporal monitoring signs. Finally, we integrated HSIC into the Actor-Critic framework to measure the independence between intraoperative temporal vital signs and its statistics.

The interpretability of our approach includes two aspects. On one hand, based on the reinforcement learning framework, we selected features just from preoperative static features and intraoperative statistical features. The selected features are the main and important features, which have a certain degree of interpretability. On the other hand, we offered post hoc explanations by focusing on instance-wise feature selection. Specifically, we employed the integral gradient method.

## 7 Conclusion and future works

We successfully applied the reinforcement learning framework to classification problems, treating feature selection as actions within this framework, which provides a novel approach. This method can not only be used for sepsis prediction but also for screening and prognosis of other diseases, as long as the problem can be considered a classification task.

This paper mainly discusses an interpretable postoperative sepsis prediction method based on a reinforcement learning-guided method coupled with HSIC. The proposed method introduced the HSIC on the basis of traditional reinforcement learning algorithms, and improved the interpretability and prediction accuracy of the model by analyzing the features of patient data. In the experiment, we used a dataset of postoperative sepsis patients from the real patients in the perioperative period of the hospital anesthesia department for training and testing. The findings reveal that our method is on par with or superior to the state-of-the-art methods, and it provides stronger interpretability. Meanwhile, our method can also effectively identify important features related to sepsis, providing more reference information for clinicians.

In summary, the proposed model provides a new ideas and methods for postoperative sepsis prediction, with high practical value and application prospects. It can provide clinical doctors with more accurate, reliable, and interpretable sepsis prediction model.

However, there are still some limitations in this method that need to be further improved and perfected in future research. Firstly, this study was conducted on a single private dataset from one institution, which may limit the generalizability of our findings. Future work should include multi-center validation using diverse external datasets to assess the model's robustness across different clinical settings and populations. Secondly, the model's predictive performance could be further enhanced by integrating multi-modal data (e.g., patient demographics, imaging data, clinical notes) with advanced feature extraction methods such as Transformer technology. Recent advances in multi-modality medical vision-language large models have demonstrated the powerful synergy between visual and textual data in clinical tasks (Li et al., 2025), suggesting that incorporating such multimodal representations could further enhance both the accuracy and interpretability of postoperative sepsis prediction. Thirdly, we can explore more efficient model training methods. In this study, we used a reinforcement learning method based on the Actor-Critic algorithm and used the HSIC for feature selection. However, in practical applications, this method may have problems such as slow convergence of training process and easy to fall into local optimum. Therefore, we can consider using other reinforcement learning algorithms and evaluation criteria to improve the efficiency and performance of model training. Finally, we can apply this method to a wider range of clinical scenarios and conduct further validation and evaluation. For example, we can consider using this method for predicting other diseases or comparing it with other prediction methods. We can also compare this method with the diagnosis results of clinicians to evaluate its feasibility and accuracy in practical applications.

In conclusion, while the proposed method demonstrates promising performance for postoperative sepsis prediction, several avenues for future research are warranted. First, to address the limitation of single-center validation, subsequent studies should focus on multi-center external evaluations using diverse datasets to establish the model's generalizability across different clinical environments and populations. Second, integrating multi-modal data, such as patient demographics, laboratory results, and medical imaging, with advanced feature extraction techniques like Transformer architectures could further enhance predictive accuracy by capturing complementary clinical information. Third, given the training efficiency challenges associated with the current Actor-Critic reinforcement learning framework, exploring alternative reinforcement learning algorithms or hybrid optimization strategies may improve convergence speed and mitigate the risk of local optima, thereby facilitating more robust and scalable clinical deployment. Fourth, prospective clinical deployment and real-time evaluation are essential to validate the practical feasibility of the proposed method within actual clinical workflows. This includes systematic comparisons with clinician judgments and further optimization of training efficiency to support timely decision-making at the point of care.

## Data Availability

The data analyzed in this study is subject to the following licenses/restrictions: The dataset contains sensitive patient information derived from perioperative electronic health records. Due to ethical approval conditions and patient privacy/confidentiality requirements, the data cannot be made publicly available and are not included in the article or supplementary materials. Requests to access these datasets should be directed to Zhenbei Liu, lzb871015@163.com.
